# Innovative 3D Microfluidic Tools for On-Chip Fluids and Particles Manipulation: From Design to Experimental Validation

**DOI:** 10.3390/mi12020104

**Published:** 2021-01-21

**Authors:** Sofia Zoupanou, Maria Serena Chiriacò, Iolena Tarantini, Francesco Ferrara

**Affiliations:** 1 CNR NANOTEC—Institute of Nanotechnology, via per Monteroni, 73100 Lecce, Italy; sophia.zoupanou@nanotec.cnr.it; 2 Department of Mathematics & Physics E. de Giorgi, via Arnesano, University of Salento, 73100 Lecce, Italy; iolena.tarantini@unisalento.it; 3 STMicroelectronics S.R.L., via per Monteroni, 73100 Lecce, Italy

**Keywords:** 3D microfluidics, lab-on-a-chip, micromixer, gradient generation, polymeric device, buried channels, particles manipulation tool

## Abstract

Micromixers are essential components in lab-on-a-chip devices, of which the low efficiency can limit many bio-application studies. Effective mixing with automation capabilities is still a crucial requirement. In this paper, we present a method to fabricate a three-dimensional (3D) poly(methyl methacrylate) (PMMA) fluidic mixer by combining computer-aided design (CAD), micromilling technology, and experimental application via manipulating fluids and nanoparticles. The entire platform consists of three microfabricated layers with a bottom reservoir-shaped microchannel, a central serpentine channel, and a through-hole for interconnection and an upper layer containing inlets and outlet. The sealing process of the three layers and the high-precision and customizable methods used for fabrication ensure the realization of the monolithic 3D architecture. This provides buried running channels able to perform passive chaotic mixing and dilution functions, thanks to a portion of the pathway in common between the reservoir and serpentine layers. The possibility to plug-and-play micropumping systems allows us to easily demonstrate the feasibility and working features of our device for tracking the mixing and dilution performances of the micromixer by using colored fluids and fluorescent nanoparticles as the proof of concept. Exploiting the good transparency of the PMMA, spatial liquid composition and better control over reaction variables are possible, and the real-time monitoring of experiments under a fluorescence microscope is also allowed. The tools shown in this paper are easily integrable in more complex lab-on-chip platforms.

## 1. Introduction

During the last decades, microfluidic structures have represented the cornerstone of common lab-on-a-chip (LOC) devices. Conventional fabrication methods include the use of standard soft lithography, followed by PDMS/PDMS, PDMS/glass, and PDMS/SU8 bonding protocols to obtain assembled microfluidic devices [1,2,3,4]. Despite the good performance of all the aforementioned molded devices, their suitability for biological studies, and the ease of process, they all are facing some limitations, which makes them ineligible for further repeated analysis and assessment, since they do not meet the requirements of robustness and stability and do not allow (in many cases) a strong reproducibility of biological or chemical assays [5,6]. Moreover, materials like polymeric silicones and resins listed above are not suitable for industrial exploitation and inappropriate out of a research laboratory [7]. Such limitations include bond strength issues, deformability, loss of hydrophilicity, lack of long-term sealing stability, and strong evaporation phenomena [8,9,10,11]. In addition, PDMS is sensitive to exposure to some chemicals and may adsorb proteins on its surface [12,13,14]. Moreover, in the realization of more complex and three-dimensional (3D) microfluidic devices, the apposition of different layers amplifies these drawbacks, as it is difficult to maintain the alignment and the good sealing of microchannels without deforming them and then affecting microfluidic features.

Thermoplastic polymers (plastics) like poly(methyl methacrylate) (PMMA), polystyrene (PS), cyclic olefin copolymers (COC), or polycarbonate (PC) have gained increasing interest during the last decade, as they allow easy surface treatment/modification, are generally transparent and biocompatible [5] and demonstrate suitability to meet some of the industrial requirements for the LOC market [15]. In particular, thanks to their low costs, they can be used for the fabrication of disposable microfluidic chips, opening a way to a wide plethora of applications, ranging from mixing [16], sample preparation, and analytical tools [17].

On the other hand, the possibility to switch from planar to 3D microfluidics has emerged as a potential revolutionary technology due to the unique properties of these miniaturized fluidic systems for their use in cell biology and on-chip chemistry to allow innovative methods like droplet microfluidics [18] and on-chip cytofluorimetry [19]. The innovation of 3D designs has gained significant attention and has been established as an important tool for different laboratory applications [20,21,22], if the entire structure is realized in transparent materials [23] and allows the real-time observation of phenomena in different and separate microchannels at the same time in a single microscopic frame.

A particularly crucial element to shift standard assays to on-chip reactions are micromixers which have a wide range of bio-applications such as studies on living cells for medical diagnostics, nanoparticles synthesis for chemistry, and biotechnologies analysis like polymerase chain reaction (PCR) [24,25,26]. In this respect, many research groups have focused their attention on this aim. Rasouli et al., as an example, designed and studied a curved micromixer channel with obstacles, in order to create normal advection and to generate Dean vortices through standard photolithography [27]. In that case, the patterned PDMS was bonded with glass. Baccouche et al. realized another microfluidic mixing device which is applied to the creation of droplets by mixing elements of two to four different tubes [28]. In another very recent study, the authors described the design of a fluidic mixer which utilizes air bubbles to facilitate mixing [4].

To combine the needs of robustness and reproducibility with the possibility to check a complex experiment into a single device making use of 3D microfluidics, we realized a stable, transparent and plug-and-play device made of PMMA, developed into three different levels. The 3D micromilled microfluidic structure is based on a PMMA/PMMA bond realized by a low-cost and easy process recently described to obtain a circulating tumor cell (CTC) device in a planar system [17]. The resulting experimental system consists of three PMMA substrates, all obtained by a micromilling machine with the following components: (i) a downstream reservoir/mixing channel on the bottom to increase the efficiency of diffusion, (ii) an upstream curved channel section, and (iii) a layer with an inlet and an outlet on the top, holding holes for firm microtubes connection and not being affected by pressure and flow strains, without the need for additional glues or gaskets for watertight connections. The pivotal element of our device is the buried transition region which creates a connection point between two of the three vertical layers by a hole between two microchannels. Since our strategy is based on sealing patterned PMMA substrates, a critical further step during the fabrication process was the bonding of the microstructured substrates. The method applied is an innovative thermal and solvent-assisted bonding, which makes use of low temperatures, low pressures, and a very common solvent (isopropanol) to allow us to obtain strong adhesion among layers, resulting in a monolithic structure with no wake points related to the typical sealing problems of microfluidic devices. Indeed, glues, clamps, or additional luer fittings and gaskets are not necessary for a watertight seal. Moreover, no burrs, cracks, or recast layers occur during the bonding process, so neither the sealing procedure nor the transparency is affected for microscope observation. 

The realization of this tool is described in next paragraphs from CAD design, optimization by the finite element method (FEM) simulation, prototyping, and final experimental tests obtained by the injection of colored liquids and differently labelled nanoparticles. 3D microchannels were used in two-way flow directions to use the two shaped microchannels (serpentine and reservoir), and this allowed us to prove the efficiency of the device for further applications for gradient creation and mixing performance mimicking macroscale turbulence [29,30]. The chip has the potential to be a tool for mixing with application in chemistry and cell biology. In cell biology studies, such a device allows for the activation of normal fibroblasts by external stimuli to become cancer-associated fibroblasts (CAFs) [31], the mixing of CAFs and associated tumor cells for interplay investigations, and the immunomagnetic labelling of circulating tumor cells to enrich a diluted sample [32]. In biochemistry, the chip could be a valid alternative to prepare a low volume of particular drugs which need “on demand” or in-situ preparation (toxic or costly reagents) to work with droplets [33], to perform digital PCR or to study protein–nanoparticles interactions [34].

## 2. Materials and Methods

### 2.1. Materials

Fluidics and particle tracing of interconnected h-junction microchannels in three dimensions were simulated with Comsol Multiphysics 5.1 computational package (COMSOL, Inc., Burlington, MA, USA), using a Computational Fluid Dynamics CFD module and specifically “mixture model, laminar flow”. During the entire simulation, the particles’ diameters were defined at 200 nm and 1 μm.

For the fabrication of the device, we used 3 square PMMA substrates (Vistacryl CQ; Vista Optics, Gorsey Lane, Widnes, Cheshire, WA8 0RP, UK), with both the length and the width of 30 mm and a thickness of 1 mm for the substrates, which hosted micromilled channels, and a thickness of 2.5 mm for the substrate, which served for the holes.

For microchannels bonding, we used pure isopropyl alcohol (Sigma-Aldrich, St. Louis, MO, USA), and for microchannels functionalization, we followed a sequence of steps which included O_2_ plasma surface treatment and the incubation of the device with 1 mg/mL bovine serum albumin (BSA) (1%) in Phosphate Buffered Saline (PBS) buffer) (Sigma-Aldrich, St. Louis, MO, USA).

The Elveflow microfluidic setup (Elvesys, Paris, France), which is suitable for these kinds of studies, was installed for pumping the solution into the device. For image and film acquisition, we used an Axio Zoom V16 fluorescence microscope (Zeiss, Oberkochen Germany) with an Apo Z 1× objective. The eyepiece of the microscope was 10×/23Br, and the numerical aperture (NA) was 0.25. The two validation tests were performed with fluorescent polystyrene microspheres of 200 nm (green) and 1 μm (red) in size (FluoSpheres^®^ Fluorescent Microspheres, Invitrogen, Ltd. 3 Fountain Drive Inchinnan Business Park, Paisley PA4 9RF, UK) for particles mixing, and ethanol (Sigma-Aldrich, St. Louis, MO, USA) and colored ink for colored liquids mixing were used. The wavelengths of fluorescence microscope channels were set according to the nanoparticles’ provider, i.e., for green particles, the excitation and emission maxima were 505 and 515 nm, respectively), and for red particles, the excitation and emission maxima were 660 and 680 nm, respectively.

### 2.2. Computational Modeling, Fabrication, Functionalization, and Testing of the Chip

#### 2.2.1. Modeling

The first step, in the scope of this work, was to model the device. To this end, we designed a 3D network, consisting of two interconnected microchannels having two independent inlets and sharing a common outlet. The COMSOL Multiphysics was then used to simulate the fluidics and particle tracing and to evaluate the mixing response of the interconnected microchannels. For the simulation, a free tetrahedral mesh was used for the entire microchannels. For the boundary conditions, we selected identical inlet velocities/flow rates, and zero static pressure was applied at the outlet. At the channels’ walls, the applied flow condition was zero, and inside the network of channels, we added water in all domains for species transport. Furthermore, we required the flow to be incompressible, steady and with no gravitational effects at all domains of the design. As a next step, we set up all the necessary conditions for particles mixing by selecting two different inlets and a common outlet and applying particle parameters from standard polystyrene beads. We also took into consideration the drag and gravitational forces. These conditions were kept constant during the whole simulation. After setting the aforementioned parameters, we evaluated the performance of the design by firstly checking the distribution of the flow at the domains of the design, but also the velocity, the flow rate, and the pressure at the inlets, junction point, and the outlet. Finally, for particles simulations, the results for both simulations, i.e., mixing two different kinds of particles and diluting a specific number of particles, were satisfactory. The numerical model used has been validated against a real system implementation.

#### 2.2.2. Design and Fabrication

The bottom, middle and top layers with reservoir, serpentine and inlets/outlet holes respectively were designed using Solidworks CAD software ( SolidWorks Corporation, 300 Baker Avenue, Concord, MA, USA), and computer-aided manufacturing (CAM) software was used to transfer the CAD information in a machine code file for the micromilling control. The Mini-Mill/GX micromilling machine (Minitech Machinery, Norcross, GA, USA) was used to realize channels and inlet/outlet holes in the PPMA layer by a 200 μm 2-flute carbide micro end milling tool. To obtain channels with a 400 μm width, a 200 μm height, and a good surface roughness, the feed rate was set at 150 mm/min with a spindle speed of 20,000 rpm. The layers were aligned using an on-board camera.

#### 2.2.3. Sealing

In order to assemble the device and to achieve a robust bonding between the PMMA layers, it was necessary to deposit hot, pure isopropyl alcohol (70 °C) on a flat bottom substrate for 10 s with a spin coater set at 2000 rpm. As soon as the bonding was established, we used binder clips to make firm alignment and incubated it for 20 min at 60 °C, without additional pressure in order to improve the bonding.

#### 2.2.4. Functionalization

The PMMA substrates suffered from the high hydrophobicity of the surface. To mitigate this, right after assembling the PMMA slices, we treated them with O_2_ plasma, which is a known method for improving surface wettability and for increasing hydrophilicity, allowing for the easier flowing of the subsequent water-based solutions. All the steps of functionalization and sample injection were performed directly in-flow, thanks to the perfect fitting of the holes with capillary tubes, allowing for a plug-and-play way of using the device. Tubes, indeed, were firmly connected, without the need for additional glues, gaskets or clamps to ensure the watertight sealing of connections. The solutions were then flowed into the capillary tubes and channels. To attenuate any unspecific sticking of fluorescent polystyrene particles on the PMMA channels surface, we incubated the chip with a blocking buffer (1 mg/mL BSA in PBS) for 2 h at room temperature.

#### 2.2.5. Experimental Tests

To evaluate the microfluidic device, we implemented two kinds of experiments for mixing and gradient generation with both colored fluids and particles. In addition, in some cases, we used on purpose the outlet as an inlet to test the influence of different path lengths and shapes of the microchannels on the mixing capabilities of the device.

During the first series of the experiments, we used two different colored liquids, which were loaded into the capillary tubes to be injected on the top (serpentine) and the bottom (reservoir) channels simultaneously. For the second series of the experiments, we injected two kinds of particles, that is, one had a diameter of 200 nm and the other had a diameter of 1 μm. This allowed us to observe the mixing behavior of liquids and particles. Both experiments were performed at different flow rates. To deliver the sample into the device, we connected the device to the Elveflow microfluidic setup by utilizing perfectly fitting capillary tubes with micromilled shaped inlets and outlets in both cases. The Elveflow micropumping system was equipped with an OB1 base module, two MkIII+ channels for a pressure controller, and two microfluidic flow sensors. With this microfluidic system, the flow of the medium can be controlled temporally. During the experiments, the two inlets were connected with a vial containing the particles samples. Subsequently, the microchip was positioned under the microscope for monitoring, and the result was validated with real-time image acquisition.

## 3. Results

### 3.1. Verification of the Numerical Model 

**Figure 1:**
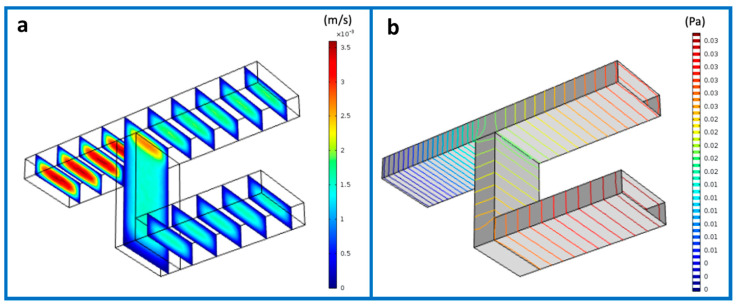
Finite element method (FEM) simulation of flow velocity (**a**) and pressure (**b**) in a two-level interconnected microchannel system.

The conceptual design of the 3D microfluidic tool has been the first step of our work. The innovative features that were required for our device led us to investigate the fluidic parameters at stake before planning the final architecture. To this end, finite element simulations were conducted to predict the performance of a 3D fluidic micromixer under two different regimes, i.e., mixing and dilution of particles. The design of the interconnected channels and the simulation results for the mixing index are given in Figure 1. In order to have a better insight on the performance of the mixing design, the flow behavior was investigated. Figure 1a reports the velocity distribution of the proposed design, showing a maximal flow at the point where the two channels meet and a minimal flow originated from the inlets till the junction point. At the channels side walls, the velocity was infinitesimal. Furthermore, Figure 1b shows the levels of the pressure at the entire network. In our design, the pressure strength was reduced from the channels connection point toward the common outlet, which was expected due to the inverse proportion to the velocity. The flow rate, which is relative to the velocity, was calculated through the given equation:

*Q* = *Aū* (1)

where *Q* is the flow rate, *A* indicates the cross-sectional area, and *ū* is the average velocity. 

**Figure 2:**
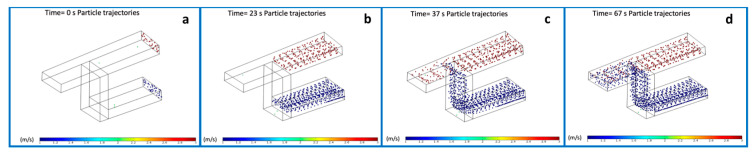
Frames of the simulation of particles mixing into three-dimensional (3D) microchannels from the starting point at t = 0 s (**a**), 23 s (**b**), 37 s (**c**) to 67 s (**d**) when particles are completely mixed.

Hence, for each experiment, i.e., mixing and diluting, we performed a sequence of simulations where we tweaked the values of some parameters in between. These parameters included the flow rate and the pressure. For the mixing experiment, a suspension of particles with sizes of 1 μm and 200 nm was injected in both inlets, respectively, and the values of flow rate ranged from 1 mL/min up to 5 mL/min between each iteration (Figure 2a–d). The resulting product was a mix of the two populations as expected in the range time of 0–67 s.

**Figure 3:**
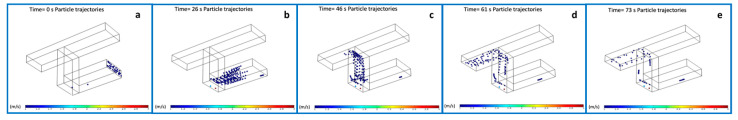
Frames of the simulation of particles dilution from the starting point at t = 0 s (**a**), 26 s (**b**), 46 s (**c**), 61 s (**d**) to 73 s (**e**).

To simulate the dilution experiment, we replaced the solution in one of the inlets with water. In Figure 3, it is reported that the particles approaching the outlet were indeed compelled to be diluted due to the presence of water on the bottom channel. Videos showing simulated behaviors for both particles mixing (Video S1) and dilution (Video S2) are provided in Supplementary Materials. 

### 3.2. Design and Fabrication of the LOC Device

**Figure 4:**
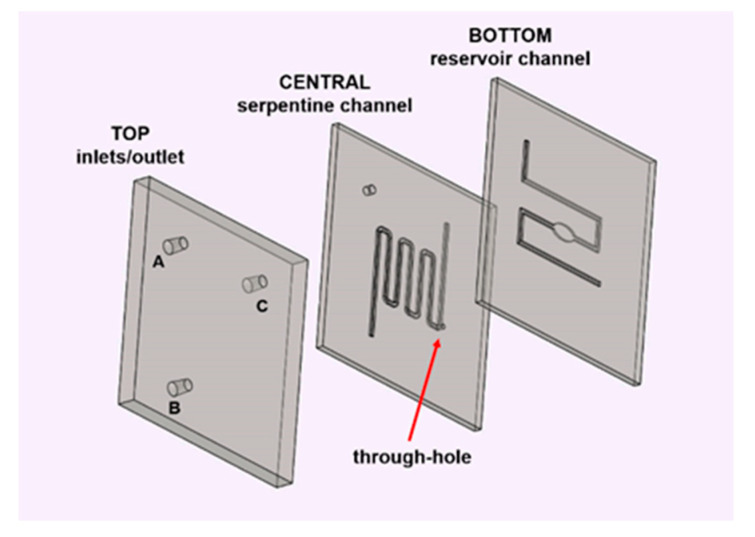
Exploded structure of the 3D microfluidic tool for the mixing and dilution of particles. The three layers (bottom, central, and top) hold the reservoir-shaped channel, the serpentine-shaped channel with a through-hole, and the inlets and outlet (indicated by A, B, and C), respectively.

In order to verify the accuracy of our numerical model in predicting the mixing process, a real mixing chip was fabricated. Selecting the proper design parameters that have the greatest influence on the mixing quality is a crucial issue for a chip made on purpose. As a first step in the realization of the device, we designed the CAD file of its elements, which was then transferred in machine code to the fabrication instruments used. Figure 4 schematically illustrates the proposed geometry. In particular, with the aim to have a structure which could be useful for both kinds of experiments, namely mixing and dilution proofs of concepts, we decided to consider a reservoir chamber in the bottom channel and a serpentine-shaped top pathway. Therefore, the entire device was constituted by a three-level structure: a reservoir-shaped channel in the first, a serpentine-shaped channel in the central, and three holes as inlets and outlet in the top layer. The three layers were separately micromilled using a mechanical micromilling machine mounting a 200 μm tool, by carefully considering alignment markers on the three layers. The length of the serpentine was of around 7 cm organized in 6 loops; the total length of the bottom channel was of around 5 cm, and the diameters of the oval reservoir were of 2 and 4 mm; the common portion ran for 1.5 cm. The substrate containing the serpentine-shaped channel held a through-hole to obtain the buried junction between the channels and to allow a 3D microfluidic pathway. The holes in the upper layer were shaped to host the capillary tubes to allow for a plug-and-play connection ensuring tight connections.

### 3.3. Bonding and Functionalization of the Device

Once the PMMA layers were fabricated, as a first step, we bonded together the substrates with microchannels; then on the top of them, we placed the slice with the holes, following the alignment scheme described above. The sealing approach used in this work was a thermal- and solvent-assisted bonding method, where hot isopropyl alcohol was spin-coated on the surfaces of the first two substrates. Then, the wet substrates were quickly aligned and firmly pressed with binder clamps. Finally, shortly after, the assembled PMMA slices were put in an oven, providing at the end of the procedure an irreversible bonding, which was solely based on this easy technique [17]. The same procedure was then reiterated to align the substrate with the holes to the former two. At the end of the procedure, the device resulted in a stable monolithic chip, with buried channels and an interconnection hole and with the inlets and outlet on the upper layer.

After assembly, the device underwent the O_2_ plasma treatment to improve hydrophilicity of the PMMA channels. Finally, we connected the device to the Elvesys micropumping system with the capillary tubes to test the bonding quality and the leakage of the chip by systematically increasing the pressure from 10 to 800 mbar while performing the monitoring via an optical microscope. We performed the in-flow functionalization with BSA to avoid polystyrene particles to stick on the channels wall and to maintain the hydrophilicity through time [35,36].

### 3.4. Mixing and Gradient Generation Experiments

With highly efficient mixing of different elements being the ultimate goal, the micromixer was tested by performing a flow test. In Table 1, a summary of the experiments we performed is reported, and the holes (A, B, and C) were used as inlets or outlets.

First and foremost, we decided to visualize the mixing behavior by using two different colored liquids. This first attempt (experiment #1 according to the scheme in Table) can reflect the fitness of the device. Flow tests were carried out using the Elveflow microfluidic setup. Once the capillary tubes were filled with the colored liquids, we injected them through the inlets, following the filling of microchannels by an Axiozoom microscope. In order to make the two fluids contemporarily reach the common portion of the channel and to obtain a chaotic particle motion at the mixing point [29], we initially set the flow parameters at 3 µL/min.

**Figure 5:**
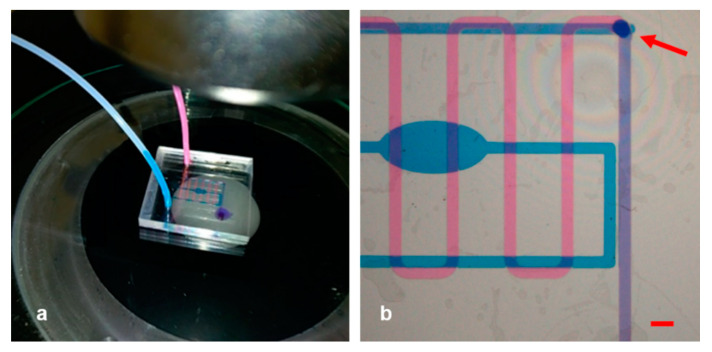
(**a**) Picture of the experimental setup for experiment #1. The blue drop emerging from the outlet of the assembled and connected device is clearly visible as a result of the mixed pink and blue fluids. (**b**) Image of the separate and common pathway taken through the microscope. Scale bar: 1 mm.

Based on different resistances and velocities of the flow (due to the different shape of the microchannels and different numbers of 90° bends of the two paths), we carefully tuned the parameters during the experiments. The long way the fluid went along, together with the large field of view of the microscope (around 10 mm) allowed us to keep eyes on the channel filling, and we reached the optimized control and mixing capabilities at a flow rate of 2.08 µL/min and a pressure of 42.80 mbar for channel 1 (serpentine) and at a flow rate of 1.65 µL/min and a pressure of 63.08 mbar for channel 2 (reservoir). The Figure 5a illustrates an overall picture of the microfluidic chip during the in-flow test we executed, in which we can observe also by naked eye that the injected pink and blue fluids resulted in a violet mixed color at the outlet. Apart from that, a more clear image obtained by the microscope is shown in Figure 5b.

The pink solution was infused in the upper serpentine channel, and the reservoir was filled with the blue one. In Figure 5b, it was vividly observable that each liquid followed its separate path and that eventually they merged at the meeting point as expected. The buried hole (indicated by the red arrow) was where the two fluids intersected and the common path began, corresponding to the very last portion of the serpentine. After that point, the two colors of the fluids were not distinguishable anymore, as they merged and turned violet.

**Figure 6:**
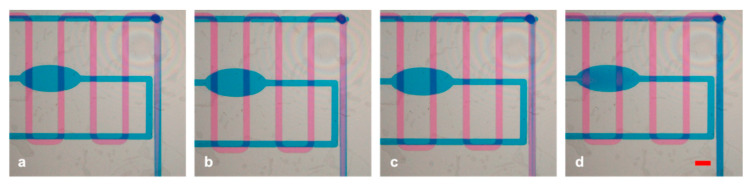
(**a**–**d**) Sequence of the pink or blue prevalence if the laminar flow is produced instead of mixing in experiment #2. Scale bar: 1 mm.

Moreover, to test the capability of the device in a laminar fluid regime, we tuned the flow rate of the channels by enhancing in turn the two channels and making the pink or blue ink prevail on the other (experiment #2, Figure 6), resulting in a gradient generator tool.

**Figure 7:**
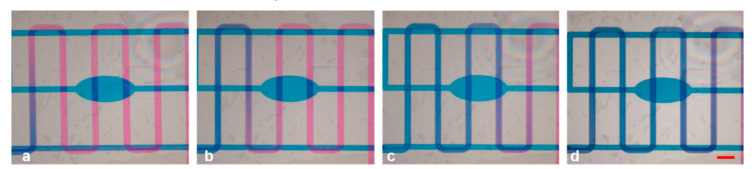
(**a**–**d**) Mixing capabilities of the serpentine-shaped channel introducing pink ink from the hole previously selected as an outlet in experiment #3. Scale bar: 1 mm.

To demonstrate the mixing capabilities of the apparatus in a longer pathway, we switched positions between one inlet and the outlet, making the serpentine channel work as a mixing channel (Experiment #3, holes A and C as inlets). In this case, we filled the serpentine channel with pink fluid injected from hole C, and we made the blue fluid arrive from the reservoir channel (hole A) to the interconnection hole, in order to reach the serpentine channel. The results are demonstrated in Figure 7, in which the pink solution in the serpentine channel started to fade after being mixed with the blue liquid and showed a transient color switch to violet. The same procedure can be applied utilizing the reservoir channel as a mixing channel (holes B and C as inlets).

**Figure 8:**
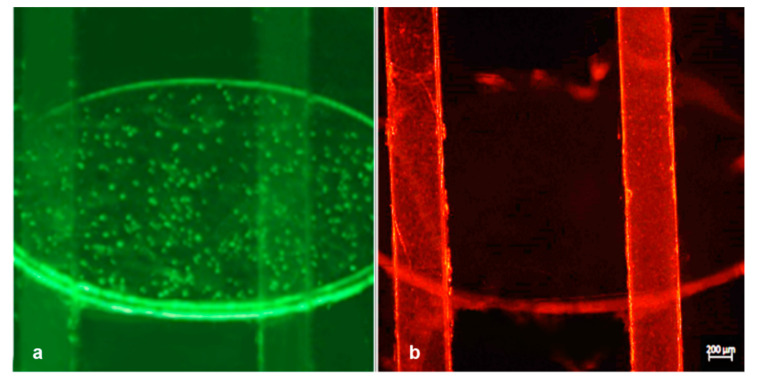
Experiment #4: merged image taken under a fluorescence microscope with subsequent fluorescence switching focused on the bottom reservoir channel (excitation/emission: 505/515 nm) filled with red nanoparticles (**a**) and the upper serpentine channel (excitation/emission: 660/680 nm) filled with red nanoparticles (**b**).

After having tested the device for watertightness and mixing capabilities, we proceeded to the second phase of the experiments with the particles mixing experiment (experiment #4). We injected a suspension of 9.1 × 10^5^/mL green fluorescent polystyrene particles of 200 nm in size to the reservoir channel through hole A and 7.2 × 10^5^/mL of 1 μm particles to the serpentine channel through hole B and recorded the normal flow with a fluorescent microscope. As can be seen, while the two flows were activated independently, the particles were clearly separated and they can be observed contemporarily in a single microscope frame. Green fluorescence can be detected only in the bottom channel, while red fluorescence was observed only in the upper one without moving the device (Figure 8).

**Figure 9:**
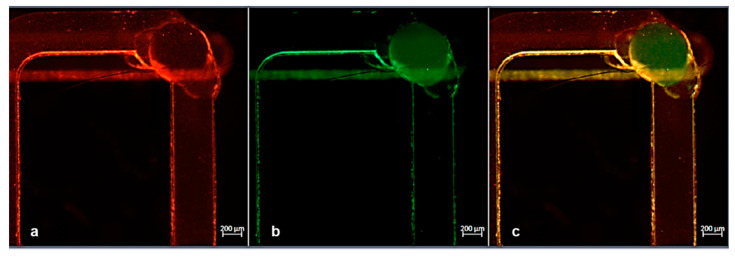
(**a**,**b**) Snapshot of the mixing point obtained by red and green fluorescence channels during experiment #4. The presence of red particles was clearly visible, while few particles were detected in the bottom and emerging flow. (**c**) Merged image of red and green channels.

It is worth noting that consecutive experiments were executed in a wide range of flow rates, in order to test the mixing performance. Since a mutually proportionate flow rate in the channels is important, each change of the flow in a channel was accompanied by a corresponding pressure compensation of the other channel, due to the higher resistance at the serpentine path. The injections of the particles were performed with a flow rate and a pressure of 2.13 µL/min and 26.42 mbar, respectively, in channel 1 (serpentine, red particles) and with a flow rate and a pressure of 1.46 µL/min and 39.52 mbar, respectively, in channel 2 (reservoir, green particles) stabilized to contemporarily reach the common portion of the channel. The path of particles was followed in real time under the microscope, and some pictures were captured in consecutive moments immediately soon before and after the mixing point (Figure 8 and Figure 9).

**Figure 10:**
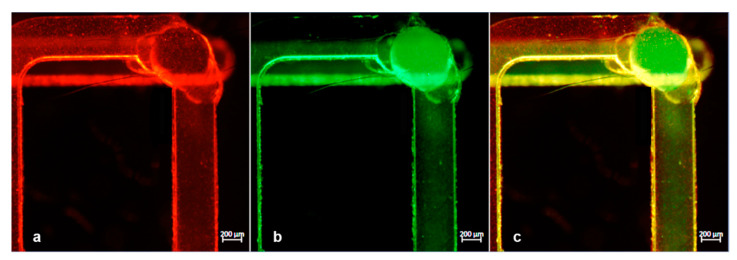
(**a**,**b**) Snapshot of the mixing point obtained by red and green fluorescence channels during experiment #4. The presence of red particles was clearly visible in the top serpentine layer, while in the bottom channel the amount of green particles was increased with respect to Figure 9b. (**c**) Merged image of the two fluorescence channels confirming the mixed particles solution in the common path.

The results of this mixing test are illustrated in Figure 9a–c, where the particles seemed to be mixed while moving from their individual channels to the common one. Figure 9a–c and Figure 10a–c were acquired by selecting separately red and green fluorescence channels with the microscope and subsequently applying the “merge” command. In this way, we can very easily distinguish both streams of particles (green and red) arriving the common channel from their individual paths.

Concretely, a comparison of Figure 9 with Figure 10 can aid to clarify that in the first, the particle mixing was just ignited since the presence of green particles was meager. In contrast, in Figure 10, we can see that a significant number of both particles were detected. This verified the mixing efficiency that needed to be met.

**Figure 11:**
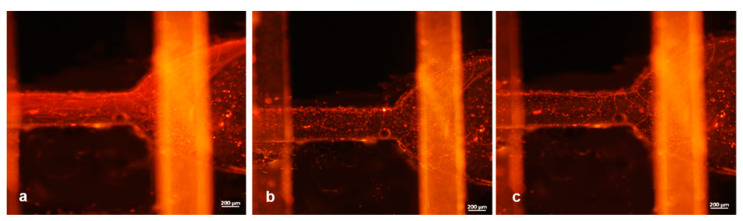
Experiment #5: on-chip particle dilution sequence. (**a**) The flow rates for particles and water injection were quite the same, and a high number of particles and associated fluorescence were clearly visible at the reservoir channel. (**b**,**c**) The flow rate of the particles was decreased, while keeping the water flow rate constant.

Further examination of the adoption of the device as a gradient generator tool was held using fluorescent particles (experiment #5). To this end, one channel was maintained with full of particles injected from the inlet of the reservoir channel (hole A); pure water was instead injected from hole C (previously considered as an outlet), running through the serpentine channel reversely. During this experiment, the same procedure was repeated to keep the water flow constant (around 7 µL/min with a pressure of 50 mbar) and gradually diminishing the particles flow between the iterations, in order to obtain a gradient generator. The values of the flow rates and the pressure for particles injection ranged from 7 µL/min (around 50 mbar) down to 1 µL/min (20 mbar) with steps of around 1.5 µL/min. Figure 11 depicts the progressive dilution of the particles. Specifically, in Figure 11a, the concentration of particles (initial concentration of 7.2 × 10^5^/mL) was at the highest level, while we kept an equal flow rate for both solutions. Subsequently, a gradual decrease of particles concentration was observed, when we tuned the flow to reach the lowest-value set (Figure 11b,c). In this way, the collected fraction emerging from the outlet gradually diluted the suspension of particles from the first to the last. The final step tested was the complete stop of the particles channel (flow 0), which led to a fraction including the particles remaining in the channel and a progressive removal of unspecific adhered polystyrene beads.

## 4. Discussion

Most major microfluidics applications demand quicker mixing, high accuracy, and low reagent volume. However, microfluidics and especially micromixing have not reached that level of efficiency yet. Recently, there has been an emergence of 3D microfluidic designs which promise to tackle these issues and streamline micromixing. Apart from that, 3D designs improve the mixing process and the possibility to perform high throughput tests: one device can be used for several parallel experiments. These are the reasons why we were encouraged to take advantage of this potential and designed and manufactured a PMMA microfluidic mixer.

In the present study, we firstly simulated the performance of an innovative 3D micromixer and then tested and ratified the modeled approach experimentally. This allowed us to identify weak points of the conceptual design and to understand how to modify the workflow in order to obtain the end-point results of our LOC tools.

In order to move into a real experimental setup, we used microfabrication by micromilling technology on PMMA substrates. Taking advantage of this highly flexible fabrication method, we were able to perfectly tune the design and to decide the best architecture for minimizing the limitations of the geometry, since it allows us to generate any feature or shape. In the right range of dimensions, micromilling has been proved to hold better performances in comparison with other fabrication methods. For example, photolithography, which is a multistep process, is almost impossible to execute without using a brand new master, which can limit the options at the prototyping phase. A device of a few square centimeters (3 × 3 cm^2^) fabricated with high precision (the channel width of 400 µm in this case), short processing time (it takes few minutes for micromilling single layers) and relatively low costs is desirable, as PMMA substrates are among the cheapest materials for the purpose. Besides its great mechanical properties, transparency, low toxicity, biocompatibility, preserved stability throughout all the experiments, and the possibility to use a well-established technique for bonding multilayers PMMA gave us also the possibility to obtain a robust and reproducible monolithic device, featuring all the characteristics required to obtain 3D microfluidics. In particular, our device consisted of three overlapped and sealed layers. The strong point of the tool is that the lowest reservoir-shaped channel and the central serpentine ran separately for the most of their course but at a certain point they were interconnected by a through-hole, resulting in the realization of a common portion of the channel, which was able to host mixing reactions. To complete the architecture of the 3D device, the third top layer was assembled to ensure the inlets and outlet connections and micromilled to perfectly fit with the tubes without the need for any additional procedures to achieve watertight experiments.

Once assembled, the device was successfully used as a mixing and dilution tool for easy integration on LOC systems. Moreover, the perfect transparency of our tool allows the contemporary observation of separate events happening in the two channels, with a single microscope observation. In the proposed configuration, the two pathways exploit the same area to run, but they are separated for the most of their lengths. The design and geometry of the channels can be easily modified on the basis of the selected application. This enables the user to perfectly monitor the inner of the channels, without moving the device or loosing the point of view to check alternatively two (or more) different behaviors. To demonstrate this, we used two differently labelled particles suspensions, injected into the two buried microchannels, and we were able to follow the two populations by simply shifting the fluorescence channel of the microscope. Mixing and gradient generation experiments were performed firstly using colored fluids to check the flow capabilities and mixing properties of the buried interconnected channels, and after we tested the mashing of the fluorescently labelled polystyrene nanoparticles, a highly efficient mixing was obtained, as demonstrated by fluorescence analysis.

In the first case, we were able to finely tune the flow and velocity of the two channels in order to obtain a complete mixing of the two colored fluids or alternatively, to maintain a laminar flow. Moreover, reversing the flow by connecting the tube from the outlet hole, we were able to make the serpentine channel as a mixing channel, in order to exploit a longer path for mixing.

After establishing flow rates and pressure conditions for colored fluids, we started dealing with particles. We injected a suspension of 9.1 × 10^5^/mL of green particles in the bottom reservoir-shaped channel and a suspension of 7.2 × 10^5^/mL of red particles in the serpentine-shaped channel. We firstly tested their presence in channels running separately till the connection hole, where the particles were forced to mix running through the common portion of the 3D microchannels. Thanks to the complete transparency of the device, we were able to follow the entire experiment under a microscope and obtain good fluorescence pictures without any interference with the PMMA multilayers.

Finally, to prove the effectiveness of our device, we provided experimental conditions for diluting particles and creating a gradient of concentration and used the bottom reservoir channel as a mixing chamber for water and particles. We injected particles and water, maintained a constant water flow and decreased the flow rate of particles from 7 µL/min to 0 µL/min with steps of 1.5 µL/min. The collected fraction from the device’s outlet was gradually more diluted over time. Based on the results of the experiments as proofs of concept, we showed that the apparatus can indeed host the expected functionality while maintaining high efficiency and accuracy.

This also proves that this LOC device’s architecture could be considered a valuable tool for a plethora of applications that require high mixing efficiency and has the potential for integration into portable systems as well. Moreover, the high stability and robustness of the assembled device make it suitable for the on-demand mixing functions (mixing and labelling cells and nanoparticles, mixing drugs which are forced to be prepared “near-the-bed”, etc.) and can enable standard microfluidic connections to be promptly merged for simple plug-and-play LOCs.

## 5. Conclusions

LOCs, defined as devices which include multiple laboratory features within a few square centimeters, have the breakthrough potential for radically changing traditional methods in various fields of chemistry, environment, and life sciences [37,38,39]. Rapid prototyping methods to design and realize polymeric LOCs are on the rise, as they allow high flexibility and precision. The fabrication of a microfluidic platform, for example, may require optimization steps in order to continuously tune the architecture and geometry on the basis of the need for handling and observation. The only way to meet these needs and allow the best experimental practice is the use of easy controllable methods of fabrication and low-cost materials. In our research activity, we optimized the realization of microfluidic tools, from the CAD design to the LOC application. In particular, we explored the possibility to obtain monolithic 3D structures with buried running channels, starting from separately microfabricated PMMA layers. In a previous study, we introduced an approach to fabricating interconnected multilevel channels for passive separation. In that work, we discussed the realization of a multilevel/3D device by using layers of LOR and SU-8 photoresists in combination with PDMS. The device, designed for biological applications, consists of two vertically stacked U-shaped channels, which share a central segment obtained by a multistep optical lithography process including a sacrificial layer modified by chemical reactions [40]. Motivated by further improving multilayer stability and fabrication processes and using materials suitable also for industrial exploitation [41], herein we demonstrate and test a novel, reusable 3D microfluidic device, in which the key characteristic is the inclusion of microfabricated PMMA substrates to obtain a structure that can work as an efficient micromixer or a gradient maker tool for LOC integration. The selection of PMMA as a rough material for LOC development derives from its suitability for milling molding and for biological applications, due to its mechanical and chemical characteristics, e.g., good thermal stability, transparency, chemical inertness, and biocompatibility. The multilayer structure has been assembled through a facile and low-cost solvent-assisted method. The obtained device showed increasing complexity and has been demonstrated to work with two different functions: mixing and obtaining of gradients into microchannels, which would take advantage of all the properties that 3D PMMA can offer. The realization of this technology expands on also the possibility of real-time observation, thanks to the complete transparency of the overlapped layers. Further improvements and envisioned applications examples would be cell labeling, a mix of two different kinds of cells in a specific ratio, or even dilution of cells for counting.

## Figures and Tables

**Table 1:** Scheme of experiments based on the alternative use of holes as inlets/outlets combined with the injection of colored fluids or fluorescent particles suspensions.

Holes	Mixing Experiment			Gradient Generation	
	Colored Fluids (#1)	Colored Fluids (#3)	Particle Suspension (#4)	Colored Fluids (#2)	Particle Suspension (#5)
**A**	in	In	in	in	out
**B**	in	Out	in	in	In
**C**	out	in	out	out	in

## Supplementary Materials

The following are available online at https://www.mdpi.com/2072-666X/12/2/104/s1, Video S1: “Particle mixing”; Video S2: “Gradient generator”.
